# The three waves in implementation of facility-based kangaroo mother care: a multi-country case study from Asia

**DOI:** 10.1186/s12914-016-0080-4

**Published:** 2016-01-27

**Authors:** Anne-Marie Bergh, Joseph de Graft-Johnson, Neena Khadka, Alyssa Om’Iniabohs, Rekha Udani, Hadi Pratomo, Socorro De Leon-Mendoza

**Affiliations:** MRC Unit for Maternal and Infant Health Care Strategies, University of Pretoria, Pretoria, South Africa; Save the Children, 2000 L Street NW, Suite 500, Washington, DC 20036 USA; Maternal and Child Survival Program, 1776 Massachusetts Avenue, NW, Suite 300, Washington, DC 20036 USA; D Y Patil University, School of Medicine, Nerul, Navi Mumbai India; Faculty of Public Health, Universitas Indonesia, Depok Campus, Depok 16424, West Java, Indonesia; Bless-Tetada Kangaroo Mother Care Foundation Phil., Inc., 7431 P. Burgos Street, San Dionisio Paranaque City Metro, Manila, Philippines

**Keywords:** Delivery of health care, Implementation, Infant premature, Kangaroo mother care, Neonatal mortality, Newborn health, India, Indonesia, Philippines

## Abstract

**Background:**

Kangaroo mother care has been highlighted as an effective intervention package to address high neonatal mortality pertaining to preterm births and low birth weight. However, KMC uptake and service coverage have not progressed well in many countries. The aim of this case study was to understand the institutionalisation processes of facility-based KMC services in three Asian countries (India, Indonesia and the Philippines) and the reasons for the slow uptake of KMC in these countries.

**Methods:**

Three main data sources were available: background documents providing insight in the state of implementation of KMC in the three countries; visits to a selection of health facilities to gauge their progress with KMC implementation; and data from interviews and meetings with key stakeholders.

**Results:**

The establishment of KMC services at individual facilities began many years before official prioritisation for scale-up. Three major themes were identified: pioneers of facility-based KMC; patterns of KMC knowledge and skills dissemination; and uptake and expansion of KMC services in relation to global trends and national policies.

Pioneers of facility-based KMC were introduced to the concept in the 1990s and established the practice in a few individual tertiary or teaching hospitals, without further spread. A training method beneficial to the initial establishment of KMC services in a country was to send institutional health-professional teams to learn abroad, notably in Colombia. Further in-country cascading took place afterwards and still later on KMC was integrated into newborn and obstetric care programs.

The patchy uptake and expansion of KMC services took place in three phases aligned with global trends of the time: the pioneer phase with individual champions while the global focus was on child survival (1998–2006); the newborn-care phase (2007–2012); and lastly the current phase where small babies are also included in action plans.

**Conclusions:**

This paper illustrates the complexities of implementing a new healthcare intervention. Although preterm care is currently in the limelight, clear and concerted country-led KMC scale-up strategies with associated operational plans and budgets are essential for successful scale-up.

## Background

Babies with a birth weight of <2500 g are the most vulnerable newborns at higher risk of mortality [1, 2]. Low birth weight (LBW) is the result of being small for gestational age (SGA) (i.e. under the 10th percentile of the reference population) [1], preterm birth (i.e. born alive before completion of 37 weeks of pregnancy) [3], or both [2]. It is estimated that 32.4 million babies were born SGA in 2010 [1], of whom nearly 15 million were preterm [4]. South and southeast Asia are the regions with the highest numbers of SGA and highest preterm birth and death rates in the world [1, 2, 4, 5]. Globally, preterm birth complications are the leading direct cause of neonatal deaths (36 %) [2] and of deaths in children under five years of age (15 %) [6].

Three-quarters of deaths from preterm birth complications are preventable without intensive care units [3]. According to an estimate by Lawn et al., kangaroo mother care (KMC) can prevent up to half of all deaths in babies weighing <2000 g at birth [7]. KMC is considered one of the most effective and scalable intervention packages for addressing high neonatal mortality pertaining to preterm births [3]. The KMC method was initiated more than 35 years ago in Colombia and has been described as ‘a rare example of a medical innovation moving from the Southern hemisphere, with recent rapid uptake in neonatal intensive care units in Europe’ [8].

Kangaroo mother care is a ‘total health-care strategy’ [9], which is applied within a supportive environment where the mother of an LBW infant is supported by healthcare workers in a healthcare facility, and by family and community members at home. The three main components of KMC are the following: skin-to-skin position against a mother’s or caregiver’s chest; exclusive breastmilk feeding as much and as long as possible; and early discharge and ambulatory care with regular follow-up visits to a healthcare facility [9–12]. KMC should be accompanied by the prevention, early recognition and appropriate management of complications [7, 13]. The practice of skin-to-skin care for more than 20 hours per day in stable infants is known as continuous KMC and is recommended as the preferred method whenever possible [9, 10].

Although strong evidence exists for the effectiveness of KMC and its components [12, 14, 15], the uptake of KMC practice and coverage of KMC services have not progressed well in many countries [16]. There is currently a global drive to investigate the reasons for this slow uptake and poor coverage. Renewed impetus for action to accelerate the scale-up of KMC has also been provided through initiatives highlighting newborn survival, such as the *Born Too Soon* report [3] and the Every Newborn Action Plan to end preventable deaths [17].

The study reported in this paper aims to contribute to a better understanding of: (a) the institutionalisation processes of facility-based KMC services at different levels of care; and (b) the reasons for the slow uptake of KMC and the scale-up of KMC services. The focus is on three countries in south and southeast Asia: India, Indonesia and the Philippines. All three rank among the top 12 countries with the largest populations in the world [18]. They are all high-burden countries with regard to LBW and preterm births and the associated morbidities and mortality [19] and fall within the 10 countries that account for 60 % of the world’s preterm births in terms of numbers [20].

The countries are among the Countdown countries for the 2015 Millennium Development Goals (MDGs), with Indonesia and the Philippines already on track in 2012 to reach or surpass their target for MDG 4 (reducing the under-five mortality rate by two-thirds between 1990 and 2015) [21]. Under-five mortality rates have been decreasing in all countries since 1990; however neonatal mortality rates have not fallen as steadily, resulting in neonatal deaths making up a large proportion of under-five deaths. Neonatal death is the cause of approximately half of the under-five deaths in these countries [22] and preterm birth complications as a cause of under-five deaths have been increasing in all countries from 2000 to 2013 [23]. The leading cause of newborn deaths in all countries is preterm birth [22]. Table 1 summarises a number of demographic and health indicators for the three countries.

**Table 1:** The three countries at a glance

Indicator	South Asia	Southeast Asia	
	India	Indonesia	Philippines
DEMOGRAPHIC INDICATORS			
Population (millions), 2013^a^	1,252.14	249.87	98.39
Life expectancy at birth, 2012^a^	66	71	69
Rank on Human Development Index, 2013^b^	135	108	117
GDP per capita (USD), 2013^a^	1,498.9	3,475.3	2,765.1
Health expenditure as % of GDP, 2012^a^	4.0 %	3.0 %	4.6 %
HEALTH INDICATORS			
Antenatal care coverage >1 visit, 2008-2012^c^	74 %	96 %	91 %
Antenatal care coverage, at least 4 visits, 2008-2012^c^	37 %	88 %	78 %
Postnatal care visits for newborn within 2 days of birth, 2008-2012^c^	--	47.8 %	--
Skilled attendant at birth, 2008-2012^c^	52 %	83 %	62 %
Institutional deliveries, 2008-2012^c^	47 %	63 %	44 %
Maternal mortality ratio, 2013^c^	190	190	120
Under-five mortality rate per 1,000 live births, 2013^c^	53	29	30
Neonatal mortality rate per 1,000 live births, 2013^c^	29	14	14
Preterm birth rate, 2010*^d^	13.0 %	15.5 %	14.9 %
Preterm births as percentage of global total, 2010^e^	23.6 %	4.5 %	2.3 %
Low birth weight rate, 2008-2012^e^	28 %	**9 %	21 %
Neonatal deaths due to preterm complications, 2013^c^	44 %	36 %	31 %
Neonatal deaths as a cause of under-5 deaths, 2013^c^	56 %	48 %	46 %
Prematurity as cause of under-5 deaths, 2013^f^	27 %	19 %	17 %

* All 3 countries are ranked under the 8 countries with the highest number of preterm births [4]

** Different time period

Data sources: ^a^World Bank, 2014 [76]; ^b^UNDP, 2014 [77]; ^c^Healthy Newborn Network, 2014 [22]; ^d^Blencowe et al. 2012 [4]; ^e^UNICEF, 2014 [20]; ^f^Liu et al. 2015 [6]

## Methods

This paper is based on an analysis and synthesis of three cross-sectional country case studies conducted in the second half of 2013 on implementation and scale-up of facility-based KMC. The Maternal and Child Health Integrated Program (MCHIP) and Save the Children’s Saving Newborn Lives (SNL) program supported the studies in India and Indonesia, and the World Health Organization Western Pacific Region (WHO-WPRO) supported the investigations in the Philippines. The studies were conducted in the ambit of health systems research and had the approval of the ministries of health of the three countries.

### Data collection

Data was collected from three main sources and focused on the state of KMC service provision:

1. Collection of background documents in the public domain that could provide insight in the state of implementation of KMC in a country (all three countries)
2. Visits to a selection of health facilities to gauge their progress with KMC implementation (India and the Philippines)
3. Interviews and/or meetings with key stakeholders as part of data collection (the Philippines) or to report on findings (Indonesia).

#### Document collection

The following types of documents were collected: government newborn-care documents; more general government documents; grey literature; conference and other meeting presentations; scientific publications; training materials; behaviour-change-communication/information-education-and-communication materials; pre- and/or in-service curricula; media documents; documents with basic newborn indicators; other relevant documents relating to newborn care produced by the United Nations (UN) and related agencies; and documents relating to the Baby-friendly Hospital Initiative or other breastfeeding endeavours.

#### Gauging the state of KMC implementation in health facilities

All countries were able to provide some information on the provision of KMC services at health facility level. In India and the Philippines a small number of facilities providing varying levels of care were visited and appraised using a standard facility questionnaire [24]. In the Philippines all 13 facilities that had received KMC training between 2003 and 2012 were visited. In India no information was available on the number of facilities providing KMC services and a convenient sample of 10 medical colleges exposed to KMC training in western and southern India between 2002 and 2004 was selected. A condensed version of the standard tool was also used in India for a self-report facility survey, with facilities identified through purposive and snowball sampling (n = 135). No facilities were visited in Indonesia, but the results from two previous studies using the same methodology and tool were considered [25, 26].

#### Stakeholder interviews and meetings

Different potentially information-rich stakeholder groups were identified for each country by means of convenient, purposive, and/or snowball sampling strategies. The stakeholder groups included: government officials and policy makers (national, provincial or state); academics and representatives of professional bodies and medical institutes; private sector activists or professionals/practitioners; development partners and donors; and service providers (functionaries) at different levels of the health system. Table 2 provides a breakdown of stakeholders consulted.

**Table 2:** Overview of stakeholders consulted in the three countries

Activities	India	Indonesia	Philippines	Total
Interviews during facility visits	40	Not applicable	15	55
Other stakeholders (individual & group interviews & meetings)	-	18	17	35
Total number of people involved	40	18	32	90

In the Philippines in-depth interviews or meetings were conducted at different levels of the health system and with donors. In India note was taken of the results of the maternal-newborn bottleneck analysis conducted with stakeholders as part of the Every Newborn Action Plan in 2013 [17]. In Indonesia the findings of a desktop review of published and unpublished materials were discussed at a stakeholder meeting in order to enhance the interpretation of existing information.

### Limitations of the study

The findings of the individual country case studies are broad brush strokes on a complex canvas made up of different country contexts and political landscapes. The range of country contexts necessitated some diversification in scope and design of the case studies and in the data collection parameters, depending on cost and time constraints. Accessibility to key stakeholders was also restricted because of operational and time constraints, travel distances, availability of informants, and the accessibility of government officials and other informants. Because of the limited focus on facility-based KMC, the country studies did not include large-scale surveys on the views of the users or potential users of KMC services or of communities in general. Furthermore, this paper does not focus primarily on barriers to and enablers of KMC practice, but rather on the processes involved in the implementation of KMC services.

## Results

In all countries the establishment of KMC services at individual facilities started before newborn, preterm and LBW care (including KMC) was officially prioritised for scale-up. Three major themes emerged from the analysis: pioneers of facility-based KMC; patterns of KMC knowledge and skills dissemination; and uptake and expansion of KMC services in relation to global trends and national policies.

### Pioneers of facility-based KMC

Awareness of the importance of newborn thermal care was first raised in the early 1990s, when countries such as Indonesia and India participated in meetings and studies coordinated by WHO-affiliated institutions and others. During this period, KMC also became more visible in the global neonatal arena, with larger randomised controlled trials [27, 28] and the broader dissemination of results on various aspects of KMC practice or specific components of the intervention. The reasons why these three countries became involved in advocacy for KMC and the dissemination of KMC knowledge and skills, while others did not, appear arbitrary. Involvement has apparently depended largely on whether people potentially interested in pursuing the initiative happened to be present at certain meetings. The first international Workshop on Kangaroo Mother Care held in Trieste, Italy, in 1996 and the second Workshop held in Bogota, Colombia, in 1998 were attended by representatives from Indonesia [29, 30]. Table 3 gives an overview of the initial activities related to KMC awareness and introduction during the period 1997 to 2001. After initial exposure, the concept of and experience with KMC were shared in professional forums and meetings in the countries.

**Table 3:** Introduction of KMC in the late 1990s

Year	Country	Activity
CONFERENCES		
1996	Indonesia	10^th^ National Congress of Indonesian Pediatricians (KONIKA), Bukit Tinggi, West Sumatra
1997	Indonesia	6^th^ National Congress Perinasia and International Symposium, Manado, North Sulawesi
1998	Philippines	National Convention of the Perinatal Association of the Philippines
1998	Philippines	Conference of the International Confederation of Midwives, Manila
2001	Philippines	Philippine Pediatric Society
WORKSHOPS		
2000	Indonesia	3^rd^ KMC International Workshop, Yogyakarta (hosted by Perinasia)
2002	India	KMC workshop for neonatologists from various states at the All India Institute of Medical Sciences (AIIMS)
INTRODUCTION OF KMC SERVICES		
1995	India	Byramjee Jeejeebhoy Medical College and Civil Hospital, Ahmedabad, Gujarat
1995/6	Indonesia	Dr Sardjito General and Teaching Hospital, Yogyakarta
1999	Philippines	Dr Jose Fabella Memorial Hospital, Manila
1999	Indonesia	Mataram General Hospital, West Nusa Tenggara Province

Visionary healthcare professionals in a few facilities began implementing KMC at their own hospital after having been exposed to KMC at an international meeting or conference, sometimes with additional orientation or training. The first hospitals in the three countries were tertiary hospitals. The Indonesian hospital was part of a three-country randomised trial to compare the effectiveness, feasibility, acceptability and cost of KMC versus conventional methods of care [27, 31].

Regarding coverage of facility-based KMC services in the Philippines in 2013, eight of the 13 hospitals that had received training in the previous decade were able to demonstrate evidence of KMC practice. By the end of 2014, KMC had been expanded to 27 more facilities in 14 of the country’s 17 regions [32]. Because of the decentralised nature of the diffusion of KMC in India and Indonesia, no official figure on the number of facilities providing KMC services was available in 2013.

### Patterns of KMC knowledge and skills dissemination

One type of training activity beneficial for enabling the initial establishment of KMC services in a country was the phenomenon of ‘institutional teams learning abroad’, i.e., training or study tours to allow teams of health professionals to visit other countries noted for their institutionalisation of KMC at specific institutions. Successful implementation on return depended on a supportive institutional environment, but did not guarantee further spread of the practice beyond the health professionals’ institution(s). The best known learning-abroad training was offered at Bogota, Colombia, with the support of the *Fundación Canguro* (Kangaroo Foundation). Two participants from one hospital (preferably a doctor-nurse team) were immersed in all the different facets of KMC practice and services for a period of three to four weeks. This training was undertaken by six teams from India between 1999 and 2003; one team each from the Philippines and Indonesia attended in 1999. Three Indonesian teaching hospitals each sent a team on a two-week South African study tour in 2008 [33].

#### Cascading of KMC training

KMC training was cascaded in the different countries by means of different types of training. Initially, when the concept was still new, stand-alone training courses and workshops were developed. Some of these are still being held.

In all three countries training and implementation were accompanied by the development of educational materials (print and video), records, checklists and other tools for use with KMC or in which KMC features. Table 4 provides a summary of specific KMC materials developed.

**Table 4:** KMC materials developed in the different countries

Country	Institution	Materials and records
India	All India Institute of Medical Sciences (AIIMS) and partners	• India KMC website (http://kmcindia.org/) • Posters • Pamphlet with KMC guidelines • Video
	King Edward Memorial (KEM) Hospital & Seth Gordhandas Sunderdas Medical College	• Manual of training of trainers • Video refined • Booklets in different languages for health personnel and community
	Individual hospitals	• Specific records and KMC charts
Indonesia	Indonesia Health Services Program	• Posters and flipcharts • Videos
	Perinasia	• Video, booklet • Training materials
	Individual hospitals	• Adapted available material from elsewhere • Developed own material
Philippines	Bless-Tetada KMC Foundation	• Manual for training of trainers • Manual for program implementers • Flipcharts for client education • KMC orientation manual for service providers • Video • Advocacy paper

India held a number of initial training drives from 2002 to 2006 with the support of Save the Children. One training initiative was coordinated from the King Edward Memorial Hospital in Mumbai for western and southern India. It included awareness and skill-based workshops, on-site training workshops (2–5 days) and one-week in-service fellowships for doctor-nurse teams. One-day, stand-alone KMC workshops have been on-going.

In Indonesia the Indonesian Society for Perinatology (Perinasia) has been training health workers in KMC since 1997 in 2-5-day workshops that have been presented in 17 cities covering 16 provinces. By the end of 2013, Perinasia had conducted 73 training courses reaching 2,190 health professionals [34]. After the establishment of a national KMC Working Group in 2009 [35], the Indonesian Ministry of Health also conducted three batches of regional KMC training courses for health personnel. Training of facility-based health workers focused on healthcare provider competencies in applying KMC and did not always appear to include sufficient focus on adult learning principles and communication skills for motivating the family to continue with KMC.

KMC training in the Philippines developed in two stages. After in-house training of staff in the Dr Jose Fabella Memorial Hospital, the Department of Health supported the training of seven more facilities in Manila in 2003. The next training drive started in 2008 with the establishment of the Bless-Tetada Kangaroo Mother Care Foundation Phil., Inc., a duly registered non-government organisation (NGO) operating in the country primarily for the scale-up and national adoption of KMC. A more comprehensive approach to training was initiated, focusing not only on the technical aspects of KMC practice but also on KMC program implementation, empowered by health care values that promote effective teamwork and administrative support. After the initial training of a core group of staff members of a regional health centre, there is a period of supportive supervision and internal cascading of training for accreditation as a centre of excellence for a specific region. An accredited centre is expected to cascade the program to the community it serves and to other health facilities in the region by conducting its own training, and monitoring and evaluation activities. Hospitals enter into a partnership with the Foundation under a three-year memorandum of agreement, subject to renewal as both parties may desire. After accreditation, the Foundation continues to support these centres through monitoring and evaluation and research to sustain quality implementation. The process in the Philippines is an example of a successful continuous public-private KMC partnership.

#### Integration of KMC into newborn-care training

Over time the KMC concept began to be integrated into other newborn care in-service training as part of essential newborn and child care and/or obstetric care, reproductive health or safe motherhood. In many instances, however, the content was not called ‘kangaroo mother care’, but rather included elements related to KMC, such as breastfeeding of the LBW infant and skin-to-skin care for thermal protection. This was the way the KMC concept was incorporated into newborn policies and guidelines in the different countries [36–39]. Unfortunately sufficient information was not available on the scope and quality of the training in the various countries, for KMC either as stand-alone training or as part of other relevant training programs.

In India training programs such as *Navjaat Shishu Suraksha Karayakram* (essential newborn care and resuscitation), Facility-Based Newborn Care, and Facility-Integrated Management of Neonatal and Childhood Illnesses contain elements of KMC. Although KMC was included in the New Policies and Protocol on Essential Newborn Care in 2009 (AO 2009–0025) [39] in the Philippines it was only incorporated as an orientation lecture in the Basic Emergency Obstetrics and Newborn Care Course.

#### Funding for training

Funding sources for KMC training varied, with contributions from individual facilities and health authorities (from local to national) in some countries, especially where KMC training is integrated into ENC and similar training programs. There were also contributions from NGOs in the Philippines and Indonesia, and from commercial companies in India and the Philippines. In the case of larger scale, stand-alone KMC training and implementation programs, global donors were involved, such as Save the Children/SNL (supported by the Bill & Melinda Gates Foundation), MCHIP (supported by USAID) and John Snow Institute, Inc. (JSI) (supported by USAID). UN agencies such as the WHO, UNICEF, and the United Nations Population Fund (UNFPA) also contributed in different ways to the development of materials for ENC and similar programs and conducted training in some countries. Training contributions from professional associations appear to have been less visible or formalised, except in the case of Perinasia, which functions as an NGO in Indonesia.

### Uptake and expansion of KMC services in relation to global trends and national policy frameworks

Despite awareness and orientation programs in KMC and fairly wide coverage of training in some countries, with individual hospitals starting implementation, the expansion of KMC services has taken off slowly in all countries. There has been a gap between implementation of KMC by the first hospital(s) and the further expansion of KMC services to other health facilities. In most cases there has been no significant spread without systematic donor or NGO input.

In each of the three countries a number of hospitals were selected to become centres of excellence to serve as training centres and centres of outreach to other hospitals in the region. In India three hospitals were identified for development in 2002. In the Philippines the Dr Jose Fabella Memorial Hospital had first been established as a centre of excellence in 2000 and was formally accredited by the *Fundación Canguro* and the Bless-Tetada KMC Foundation in 2011, with two further centres accredited by 2012. In Indonesia three hospitals were established as centres of excellence in 2008/9. Only the hospitals in the Philippines are linked to a longer term system of support and eventual KMC accreditation by a national KMC-dedicated NGO. In Indonesia possible ways of linking KMC with the revised Standards for Hospital Accreditation [40] are being discussed.

Commitment to KMC implementation and the drafting of guidelines or standard operating procedures (SOPs) at facility level was evident in some of the hospitals visited during the study. The reason why these hospitals could not achieve large-scale expansion to other facilities may be that national scale-up plans or coordination mechanisms were not in place by 2013. National commitment to KMC implementation often only becomes visible when KMC is included in national maternal and/or newborn policy frameworks [24]. The Indonesian Ministry of Health did, however, establish a multi-professional National KMC Working Group by ministerial decree in 2009 [35] to provide guidance on the further development of the KMC program with regard to policies, standards and regulations. None of the countries had any systematic national reporting mechanisms for the progress of KMC implementation or for reporting on indicators related to the provision of KMC services (e.g. coverage: number of total of LBW babies receiving KMC; impact: mortality and morbidity figures for preterm and LBW babies receiving KMC compared to those not receiving it). KMC facilities established in the Philippines under the KMC Foundation submit data to the Foundation. Facility administrations receive annual feedback and this report is used as a benchmark for each facility when planning its next steps in quality improvement and expansion to surrounding communities.

#### Waves of KMC uptake

In all countries with KMC services there appear to have been three waves of uptake of KMC in facility-based services, albeit taking different forms. In every country the first wave coincided with the years around the millennium and the second wave with the years 2007 to 2012. We are currently seeing a third wave with a global push for implementing cost-effective and high-impact newborn interventions, inter alia through the Every Newborn Action Plan [41] and the drive for the acceleration of KMC as demonstrated in the Istanbul Declaration on Kangaroo Mother Care Acceleration [16]. Table 5 provides an overview of government policy and KMC implementation trends across the three waves in the three countries.

**Table 5:** Waves of KMC expansion in the three countries

Country	Policy and implementation	Wave 1 (1998–2006)	Wave 2 (2007–2012)	Wave 3 (2013+)
*SHIFT IN INTERNATIONAL FOCUS:*		• Focus: Infant and child survival • Strategy: Scale-up of effective proven, low-cost preventive care and treatment for diseases contributing to under-five mortality	• Focus: Newborn survival • Strategy: Continuum of care approach: institutionalisation of essential newborn care (ENC)	• Focus: Preterm and LBW survival • Strategy: Global acceleration of KMC as a high-impact intervention
India	Government policies and initiatives	-	• *Janani Shishu Suraksha Karyakram* (complete care for mother-baby dyad) • *Navjaat Shishu Suraksha Karayakram* (ENC)	• Neonatal Task Service Group (2013) – development of KMC guidelines • KMC integrated into the India Newborn Action Plan (INAP) (2014)
	KMC implementation	• 3 centres of excellence introduced KMC services in 2000 • Training initiatives (2002–6)	• Half the facilities that responded to the self-report survey introduced KMC after 2009	• Revitalisation after the Ninth International KMC Conference in Ahmedabad (Nov 2012) • Introduction and implementation of KMC in all districts of Tamil Nadu state (2013)
Indonesia	Government policies and initiatives	-	• Indonesian Neonatal Working Group: set goal of introducing KMC as the standard protocol for LBW care in up to five teaching hospitals within two years (2007) • National KMC Task Force Team (2009) • KMC integrated into the Mother and Baby Friendly Hospital (RSSIB) guidelines (2009) • Guidelines for in-hospital care of the LBW infant (2009) • Revised Standards for Hospital Accreditation (2012)	• KMC integrated into the Indonesian Newborn Action Plan (INAP) (2014)
	KMC implementation	• Third International KMC Workshop (2000) • 2 centres for practical training • Coordination of training by Perinasia	• 3 centres of excellence (2008/9) • Support for 10 hospitals (2009–10) • Support for 3 hospitals (2011) • Selected community health centres (Garut district) (2009) • Seminar and workshop, Gajah Mada University, Yogyakarta (2009) • Continued training by Perinasia	• Further attempts to institutionalise KMC
Philippines	Government policies and initiatives	-	• New policies and protocol on essential newborn care (2009) – close collaboration with Safe Motherhood • ‘First Embrace’ (essential intrapartum newborn care) (2012)	• Initiation of the Program of Care for the Small Baby that includes the full KMC package (2015)
	KMC implementation	• 1 centre of excellence with 1 city-wide network on KMC through local government unit (Manila)	• 2 additional centres of excellence with 3 more being developed (Bless-Tetada KMC Foundation)	• 30 neonatologists trained as trainers by the Bless-Tetada KMC Foundation • Accreditation of a further 2 centres of excellence • Development of 10 more centres through the Bless-Tetada KMC Foundation • 5 other centres being developed through Dr Jose Fabella Memorial Hospital • From 2013 to 2015: - Expansion from 13 to 51 facilities with KMC services (44 public and 7 private hospitals) - Expansion from 4 to 16/17 regions • 22 facilities trained in the Program of Care for the Small Baby (2015)

#### KMC champions

During the first two waves the establishment of KMC services was driven by different champions. Neonatologists played a key role in India and the Philippines, with the KMC Foundation in the Philippines – founded by a neonatologist – acting as the driving force for the expansion of KMC. In Indonesia the process was driven by neonatologists, paediatricians and Perinasia, a professional NGO. Perinasia is a multidisciplinary organisation and members include other medical practitioners, nursing/midwifery professionals, public health specialists and psychologists with a common interest in maternal and newborn care.

Health professionals acting as advocates for KMC during the first two waves had to provide evidence of the effectiveness and safety of the method, which led to a number of published KMC studies from Indonesia [27, 31, 42–44], the Philippines [45, 46] and India [47–57].

## Discussion

One of the main findings of this study was the differences in adoption, training and implementation trajectories for facility-based KMC over a period of 20 years. In terms of Roger’s theory of diffusion of innovations [58], the first facility-based innovators were mostly neonatologists and paediatricians. They were followed by a few more early-adopting institutions in the early 2000s. By 2013, none of the countries had reached the stage where there was an early majority of facilities providing KMC services. Opinion leaders at different levels of the health system as a social system were not ready to adopt the concept across the board. It is only now when global opinion leaders are highlighting the importance of KMC that country governments have started to apply the concept in practice. The lack of consistent funding or overdependence on donor funds appears to have been largely responsible for slowing the uptake and support of KMC services.

### Trajectories of KMC implementation

The trajectory of KMC implementation in the three countries followed a similar sequence to that in other countries [24]. The first individual champions – largely neonatologists and paediatricians – demonstrated leadership by pioneering KMC, mostly in central or teaching hospitals without any immediate diffusion of the practice. This corresponds to the grassroots dimension of KMC implementation where individual institutions or individuals with an interest initiated some form of KMC practice in the neonatal intensive- or high-care unit [59]. These champions were, however, not political entrepreneurs familiar with advocacy strategies who could promote KMC on the policy agenda beyond their own hospital [60].

In India and Indonesia donor support for interventions in project mode enabled further uptake. In the Philippines an NGO acted as a catalyst for the first spread beyond the capital. The decentralised nature of the health system in all three countries may also have contributed to pockets of inertia in KMC scale-up. In Indonesia, for example, support for KMC from the central Ministry of Health did not guarantee commitment at regional or district level [33].

Difficulties in obtaining reliable data on KMC coverage and practice at a country, regional or district level have also been reported for other countries [24]. Darmstadt et al. see KMC as an emergency newborn care intervention for LBW babies in health facilities, which could be tracked by means of surveys and health management information systems, although this is currently not done [61]. The milestones and actions at national and global level proposed by Mason et al. as part of the Every Newborn initiative include KMC as an indicator in the care of small newborns. Their goal is that >50 % of these babies should receive KMC by 2020 and >75 % by 2030 [62].

### Training for kangaroo mother care

India, Indonesia and the Philippines all benefited from the ‘learning abroad’ principle and all established different forms of ‘training centres’ [59]. In India KMC implementation followed the academic path [59], with training provided by a few tertiary teaching hospitals and resources made available through a website. In Indonesia and the Philippines, a professional association and an NGO run by professionals, respectively, fulfilled the main training function. This could be explained by the fact that at the time when more systematic training was conducted with a view to further diffusing the KMC innovation, government policy focus was still on child survival with little attention to newborn and LBW care. The initial training packages tended to focus on training in the practice of KMC without sufficient focus on preparation for implementing KMC (including institutional and structural support needed) and on supportive supervision for sustainability. This is typical of training that takes place in project mode [24]. Where KMC was integrated into newborn and obstetric care training packages, the training may have been more theoretical and limited in scope [63] without providing a sense of agency of how individuals could influence and change practice that improves patient care. In other countries KMC also ‘got lost’ [64] because too many newborn interventions and improvements were required at the same time.

### The three waves of KMC implementation

The three waves of KMC implementation are linked to global trends in health focus. The first two waves correspond to Shiffman’s observations that until the 1990s the work in low-income countries was focused on infant and child survival [65], with programs such as the Integrated Management of Childhood Illnesses (IMCI) [66]. The increased focus on newborn survival started around 2005 with the publication of the Neonatal Survival Series in *The Lancet* and the increased support from agencies other than Save the Children/SNL and informal networks of professionals [65]. The focus of the first two waves may help to explain (a) why most pioneer institutions were specialised tertiary institutions not really able to facilitate large-scale expansion of KMC beyond their own institution; (b) why it was so difficult to propose and implement KMC scale-up programs until about 2010; and (c) why in all the countries in this study the coverage of KMC services was still quite poor by 2013. The third wave commenced with the publication of the *Born Too Soon* report [3] and the launch of Every Newborn Action Plan [41]. Two KMC topics were prioritised on the accompanying research agenda proposed by Yoshida et al.: ‘How can facility-based initiation of kangaroo mother care or continuous skin-to-skin contact be scaled up?’ and ‘Can community-based initiation of kangaroo mother care reduce neonatal mortality of clinically stable preterm and low birthweight babies?’ [67]. Commitment and planning for scale-up have already commenced in the three countries with the publication of Every Newborn action plans in India and Indonesia, the development of KMC guidelines in India and the introduction of ‘First Embrace’ (essential intrapartum newborn care) in the Philippines (Table 5).

### The way forward

In 2014 Lawn and colleagues stated: ‘From a position of near-invisibility, newborn survival, and particularly preterm birth, is now on national agendas, having been pulled into the limelight by the policy hook of the MDGs and improved epidemiological estimates. *Funding and action, however, are lagging* [our emphasis]’ [2]. Real national commitment, reflected by inclusion of KMC in relevant national policies, guidelines, protocols and SOPs, is one of the first critical steps in achieving effective coverage of KMC services and clinical standardisation. During the current third wave of KMC implementation – with LBW care increasingly included in the countries’ national health agendas – there may be a shift from individual health professionals as drivers and advocates of, or hindrances to, the implementation of KMC, to policy makers and administrators driving the scale-up process from a health-systems perspective. Current knowledge of the beneficial effects of KMC (generated in ‘objective, measurable and controllable ways … at a population level’ [68]) and of KMC implementation processes (generated by country case studies that ‘take account of the cultural and psychosocial context’ [68]) should be translated into action at all levels of countries’ health systems, with appropriate monitoring and evaluation of the process linked to measures of performance and accountability.

Responses in the bottleneck investigation that was part of the Every Newborn Action Plan included the absence of an investment plan for scale-up of KMC, with no funding allocation to KMC implementation, resulting in high dependency on external funding [17]. In all countries more advocacy is required to persuade financial decision makers to allocate sufficient funds in maternal, newborn and child health government budgets for KMC, while ensuring that both capital and recurring costs are considered. A review by Barasa et al. found that incentives generated by funding arrangements influence the way in which budgetary priorities are set [69]. Furthermore, assistance from development partners may be very important for initial establishment of services, training, and community and family support activities. Such endeavours should pay appropriate attention to potential tensions that could arise between stakeholders and funders in the evaluation of interventions [70]. It is, therefore, essential that donor support for KMC implementation is undertaken within a well-defined country-led scale-up strategy.

The focus of this study was limited to the provision of facility-based KMC services. It is acknowledged that almost half the preterm babies in middle- and low-income countries are born at home [3]. Although there is currently insufficient evidence with regard to community-initiated KMC for preterm babies, post-discharge KMC is recommended for reducing subsequent mortality and improving breastfeeding rates [71]. There are currently initiatives, like the Selaras Project in Indonesia, for improving health worker training in pre-discharge counselling of parents and the family to help them to understand the care and monitoring of the LBW baby at home (personal communication, P Kaslam). Globally there is also a strong drive to learn more about the potential for community-based KMC [72] and the practice of KMC in the community, as part of the broader newborn care approach [17, 73]. One of the current weaknesses in many countries is the inadequate follow-up of KMC babies after discharge from the health facility [17, 24, 74], inter alia due to poor referral linkages between different levels of the health system [33]. Any scale-up activities should emphasise the strengthening of linkages [17] between community and facility-based KMC and the inclusion of the frontline health workers (e.g. community health workers) who should be in constant contact with families throughout pregnancy and the post-partum care period [75].

## Conclusion

This paper illustrated the complexities of the implementation of a new healthcare intervention. Professional resistance and the lack of political priority for newborn and LBW care within the political and healthcare structures at the time when early adopters introduced KMC into their health facilities explain why the vast majority of institutions were left behind. Only now that KMC has been included in the global health agenda as one of the key interventions for the reduction of newborn morbidity and mortality can we expect the majority to follow suit. However, this expectation might not materialise without a country-led clear and concerted KMC scale-up strategy with an associated operational plan and budget.

Enabling mothers and their families to practice kangaroo mother care entails a complex interplay between health-system requirements, organisational culture, human behaviour and community networks. Scaling up KMC is not an easy process and it takes time for services to become institutionalised and integrated into the total newborn care package. Leadership reflecting an understanding of the necessity of prioritising the provision of facility-based KMC services and good governance at all levels throughout the implementation process is essential. Scale-up processes should be driven by strategies to ensure appropriate quality of care.

## References

[1] Black RE, Victora CG, Walker SP, Bhutta ZA, Christian P, De Onis M (2013). Maternal and child undernutrition and overweight in low-income and middle-income countries. Lancet.

[2] Lawn JE, Blencowe H, Oza S, You D, Lee ACC, Waiswa P (2014). Every Newborn: progress, priorities, and potential beyond survival. Lancet.

[3] March of Dimes, PMNCH, Save the Children, World Health Organization: Born Too Soon: The Global Action Report on Preterm Birth. Geneva: World Health Organization; 2012.

[4] Blencowe H, Cousens S, Oestergaard MZ, Chou D, Moller AB, Narwal R (2012). National, regional, and worldwide estimates of preterm birth rates in the year 2010 with time trends since 1990 for selected countries: a systematic analysis and implications. Lancet.

[5] Oza S, Lawn JE, Hogan DR, Mathers C, Cousens SN (2015). Neonatal cause-of-death estimates for the early and late neonatal periods for 194 countries: 2000–2013. Bull World Health Organ.

[6] Liu L, Oza S, Hogan D, Perin J, Rudan I, Lawn JE (2015). Global, regional, and national causes of child mortality in 2000–13, with projections to inform post-2015 priorities: an updated systematic analysis. Lancet.

[7] Lawn JE, Mwansa-Kambafwile J, Horta BL, Barros FC, Cousens S (2010). ‘Kangaroo mother care’ to prevent neonatal deaths due to preterm birth complications. Int J Epidemiol.

[8] Lawn JE, Davidge R, Paul VK, Von Xylander S, De Graft JJ, Costello A (2013). Born Too Soon: Care for the preterm baby. Reprod Health.

[9] Nyqvist K, Anderson C, Bergman N, Cattaneo A, Charpak N, Davanzo R (2010). Towards universal Kangaroo Mother Care: recommendations and report from the First European conference and Seventh International Workshop on Kangaroo Mother Care. Acta Paediatr.

[10] Charpak N, Ruiz J, Zupan J, Cattaneo A, Figueroa Z, Tessier R (2005). Kangaroo Mother Care: 25 years later. Acta Paediatr.

[11] Nyqvist K, Anderson C, Bergman N, Cattaneo A, Charpak N, Davanzo R (2010). State of the art and recommendations: Kangaroo mother care: application in a high-tech environment. Acta Paediatr.

[12] Ruiz JG, Charpak N (2007). Evidence-based clinical practice guidelines for an optimal use of the kangaroo mother method in preterm and/or low birthweight infants at birth.

[13] Maternal and Child Health Integrated Program (MCHIP) (2012). Kangaroo mother care implementation guide.

[14] Conde-Agudelo A, Diaz-Rossello J (2014). Kangaroo mother care to reduce morbidity and mortality in low birthweight infants. Cochrane Database Syst Rev.

[15] Ludington-Hoe SM, Morgan K, Abouelfettoh A (2008). A clinical guideline for implementation of kangaroo care with premature infants of 30 or more weeks’ postmenstrual age. Adv Neonatal Care.

[16] Engmann C, Wall S, Darmstadt G, Valsangkar B (2013). Claeson M, on behalf of the participants of the Istanbul KMC Acceleration Meeting: Consensus on kangaroo mother care acceleration. Lancet.

[17] Dickson E, Simen-Kapeu A, Kinney MV, Huicho L, Vesel L, Lackritz E (2014). Every Newborn: health-systems bottlenecks and strategies to accelerate scale-up in countries. Lancet.

[18] United States Census Bureau: International programs: country rank; 2013. [http://www.census.gov/population/international/data/countryrank/rank.php].

[19] Lee ACC, Katz J, Blencowe H, Cousens S, Kozuki N, Vogel JP (2013). National and regional estimates of term and preterm babies born small for gestational age in 138 low-income and middle-income countries in 2010. Lancet Glob Health.

[20] United Nations Children’s Fund (UNICEF) (2014). State of the World’s Children.

[21] United Nations Children's Fund (UNICEF): Promise renewed progress summary; 2012. [http://www.unicef.org/esaro/Promise_renewed_progress_summary.pdf].

[22] Healthy Newborn Network: Newborn numbers (November 13, 2014); 2014. [http://www.healthynewbornnetwork.org/page/newborn-numbers].

[23] World Health Organization: Mortality and global health estimates for causes of child death by country; 2015. [http://apps.who.int/gho/data/node.main.ghe300-by-country?lang=en].

[24] Bergh A-M, Kerber K, Abwao S, De-Graft Johnson J, Aliganyira P, Davy K (2014). Implementing facility-based kangaroo mother care services: lessons from a multi-country study in Africa. BMC Health Serv Res.

[25] Pratomo H, Rustina Y, Poernomo Sigit Sidi I, Uhudiyah U, Suradi R, Handayani S: Pengembangan Program Pelayanan PMK di Indonesia. Catatan Evaluasi Pengalaman Perinasia dalam Pengembangan Program PMK di Indonesia, Perkumpulan Perinatologi Indonesia (Perinasia) [Development of the Kangaroo Mother Care program in Indonesia. A note on the evaluation of the development of KMC services by the Indonesian Society for Perinatiology (Perinasia)]. Jakarta: Perinasia; 2012.

[26] Bergh A-M, Rogers-Bloch Q, Pratomo H, Uhudiyah U, Poernomo Sigit Sidi I, Rustina Y (2012). Progress in the implementation of kangaroo mother care in ten hospitals in Indonesia. J Trop Ped.

[27] Cattaneo A, Dvanzo R, Worku B, Surjono A, Echeverria M, Bedri A (1998). Kangaroo mother care for low birthweight infants: a randomized controlled trial in different settings. Acta Paediatr.

[28] Charpak N, Ruiz-Peláez JG, Figueroa Z, Charpak Y (1997). Kangaroo mother versus traditional care for newborn infants ≤2000 grams: a randomized, controlled trial. Pediatrics.

[29] Cattaneo A, Davanzo R, Uxa F, Tamburlini G, for the International Network on Kangaroo Mother Care (1998). Recommendations for the implementation of Kangaroo Mother Care for low birthweight infants. Acta Paediatr.

[30] Charpak F, Figueroa Z, Ruiz JG, on behalf of the participants of the Second International Workshop on Kangaroo Mother Care (2000). ”The Bogota Declaration on KMC”: conclusions at the second international workshop on the method. Acta Paediatr.

[31] El H, Surjono A, Setyowireni D (2002). Kangaroo-mother care in low birth weight infants: a randomized controlled trial. Paediatr Indones.

[32] De Leon-Mendoza S (2014). Status of kangaroo mother care programs in the Philippines.

[33] Pratomo H, Uhudiyah U, Poernomo Sigit Sidi I, Rustina Y, Suradi R, Bergh A-M (2012). Supporting factors and barriers in implementing kangaroo mother care in Indonesia. Paediatr Indones.

[34] Tobing H: Kompilasi Data Pelatihan Perawatan Metode Kanguru oleh Perinasia [Compilation of Kangaroo Mother Care Training by Perinasia]. Unpublished manuscript; 2013

[35] Indonesia Ministry of Health (2009). Surat Keputusan Menkes RI No: 203/Menkes/SK/III/2008 tentang Pembentukan Kelompok Kerja Nasional Perawatan Metoda Kanguru [Decree of the Ministry of Health Number: 203/MOH, SK/III/2009 on the Establishment of the National Working Group on KMC].

[36] India Ministry of Health and Family Welfare (2013). A Strategic Approach to Reproductive Maternal, Newborn, Child and Adolescent Health (RMNCH + A) in India.

[37] Indonesia Ministry of Health: Pedoman Pelaksanaan Program Rumah Sakit Sayang Ibu dan Bayi [Guidelines for the Mother Baby Friendly Hospital Program]. Jakarta: Ministry of Health, Directorate General for Medical Services; 2009.

[38] Indonesia Ministry of Health (2009). Pedoman Peyanan Kesehatan Bayi Berat Lahir Rendah (BBLR) dengan Perawatan Metoda Kanguru di Rumah Sakit dan Jejaringnya [Guideline for the caring the low birth weight babies using KMC in the hospital and its network.

[39] Philippines Department of Health (2009). Adopting New Policies and Protocol on Essential Newborn Care. Administrative Order 2009–0025.

[40] Indonesia Ministry of Health: Standar Akreditasi Rumah Sakit [Standard Hospital Accreditation]. Jakarta: Ministry of Health, Directorate General for Medical Services, Hospital Accreditation Committee; 2012.

[41] Samarasekera U, Horton R (2014). The world we want for every newborn child. Lancet.

[42] Suradi R, Chair I, Thaha RM (1998). Acceptance of the kangaroo care method by mothers in rural areas. Paediatr Indones.

[43] Eka Pratiwi IGAP, Soetjiningsih, Made Kardane I (2009). Effect of kangaroo method on the risk of hypothermia and duration of birth weight regain in low birth weight infants: a randomized controlled trial. Paediatr Indones.

[44] Alisjahbana A, Usman A, Trijati SI (1998). Prevention of hypothermia of low birth weight infants using Kangaroo Mother Care method. Pediatr Indones.

[45] De Leon-Mendoza S: Impact of kangaroo mother care (KMC) on the survivability of the moderately-low birth weight neonate. Dr Jose Fabella Memorial Hospital Med J 2001 2:1

[46] Ballesteros RM, Agulay EA, Dagdag-Matias AA, De Leon-Mendoza S (2013). Kangaroo mother care: a randomized controlled trial on its effects on growth and stability among low birth weight infants ≤ 2000 grams in a tertiary government hospital. Philippine J Ped.

[47] Ramanathan K, Paul VK, Deorari AK, Taneja U, George G (2001). Kangaroo Mother Care in very low birth weight infants. Indian J Ped.

[48] Kadam S, Binoy S, Kanbur W, Mondkar JA, Fernandez A (2015). Feasibility of kangaroo mother care in Mumbai. Indian J Ped.

[49] Gupta M, Jora R, Bhatia R (2007). Kangaroo mother care (KMC) in LBW infants – a western Rajasthan experience. Indian J Ped.

[50] Suman RP, Udani R, Nanavati R (2008). Kangaroo mother care for low birth weight infants: a randomized controlled trial. Indian Ped.

[51] Gathwala G, Singh B, Balhara B (2008). KMC facilitates mother baby attachment in low birth weight infants. Indian J Ped.

[52] Gathwala G, Singh B, Singh J (2010). Effect of Kangaroo Mother Care on physical growth, breastfeeding and its acceptability. Trop Doct.

[53] Kumar V, Mohanty S, Kumar A, Misra RP, Santosham M, Awasthi S (2008). Effect of community-based behaviour change management on neonatal mortality in Shivgarh, Uttar Pradesh, India: a cluster-randomised controlled trial. Lancet.

[54] Ali SM, Sharma J, Sharma R, Alam S (2009). Kangaroo mother care as compared to conventional care for low birth weight babies. Dicle Med J.

[55] Parmar VR, Kumar A, Kaur R, Parmar S, Kaur D, Basu S (2009). Experience with kangaroo mother care in a neonatal intensive care unit (NICU) in Chandigarh. Indian J Ped.

[56] Ghavane S, Murki S, Subramanian S, Gaddam P, Kandraju H, Thumalla S (2012). Kangaroo Mother Care in Kangaroo ward for improving the growth and breastfeeding outcomes when reaching term gestational age in very low birth weight infants. Acta Paediatr.

[57] Udani RH, Kabra N, Nanavati RN, Aloke VR (2013). Kangaroo Mother Care (KMC): A cohort follow up study on impact of duration of KMC on mortality, morbidity, hospital stay and breast feeding. J Neonatol (India).

[58] Rogers E (1995). Diffusion of innovations.

[59] Bergh A-M, Charpak N, Enzeonodo A, Udani R, VanRooyen E, for the KMC Education and Training Working Group (2012). Education and training in the implementation of kangaroo mother care. S Afr J Child Health.

[60] Shiffman J, Sultana S (2013). Generating political priority for neonatal mortality reduction in Bangladesh. Am J Public Health.

[61] Darmstadt GL, Kinney MV, Chopra M, Cousens S, Kak L, Paul VK (2014). Lawn JE, for The Lancet Every Newborn Study Group: Who has been caring for the baby?. Lancet.

[62] Mason E, McDougall L, Lawn JE, Gupta A, Claeson M, Pillay Y (2014). From evidence to action to deliver a healthy start for the next generation. Lancet.

[63] Aliganyira P, Kerber K, Davy K, Gamache N, Sengendo NH, Bergh A-M (2014). Helping small babies survive: an evaluation of facility-based Kangaroo Mother Care implementation progress in Uganda. Pan Afr Med J.

[64] Bergh A-M, Manu R, Davy K, Van Rooyen E, Quansah Asare G, Awoonor Williams JK (2012). Translating research findings into practice – the implementation of kangaroo mother care in Ghana. Implement Sci.

[65] Shiffman J (2010). Issue attention in global health: the case of newborn survival. Lancet.

[66] Bryce J, Victora CG, Habicht JP, Black RE, Scherpbier RW, on behalf of the MCE-IMCI Technical Advisors (2005). Programmatic pathways to child survival: results of a multi-country evaluation of Integrated Management of Childhood Illness. Health Policy Plan.

[67] Yoshida S, Rudan I, Lawn JE, Wall S, Souza JP, Martines J (2014). Newborn health research priorities beyond 2015. Lancet.

[68] Shaw RL, Larkin M, Flowers P (2014). Expanding the evidence within evidence-based healthcare: thinking about the context, acceptability and feasibility of interventions. Evid Based Med.

[69] Barasa EW, Molyneux S, English M, Cleary S (2015). Setting healthcare priorities in hospitals: a review of empirical studies. Health Policy Plan.

[70] Bates I, Boyd A, Aslanyan G, Cole DC (2015). Tackling the tensions in evaluating capacity strengthening for health research in low- and middle-income countries. Health Policy Plan.

[71] Bhutta ZA, Das JK, Bahl R, Lawn JE, Salam RA, Paul VK (2014). Can available interventions end preventable deaths in mothers, newborn babies, and stillbirths, and at what cost?. Lancet.

[72] Conde-Agudelo A, Diaz-Rossello J: Kangaroo mother care to reduce morbidity and mortality in low birthweight infants. Cochrane Database Syst Rev 2011 (Issue 3: Art. No. CD002771). 10.1002/14651858.CD002771.pub2 21412879

[73] Knippenberg R, Lawn JE, Darmstadt GL, Begkoyian G, Fogstad H, Walelign N (2005). Systematic scaling up of neonatal care in countries. Lancet.

[74] De Leon-Mendoza S, Mokhachane M (2011). “Early” or timely discharge in kangaroo mother care: evidence and experience. Current Women’s Health Rev.

[75] Darmstadt GL, Marchant T, Claeson M, Brown W, Morris S, Donnay F (2013). A strategy for reducing maternal and newborn deaths by 2015 and beyond. BMC Pregnancy Childbirth.

[76] The World Bank: Indicators (Health); 2014. [http://data.worldbank.org/indicator/].

[77] United Nations Development Programme (UNDP): Human development reports; 2014. [http://hdr.undp.org/en/content/human-development-index-hdi-table].

